# Understanding clinical prediction models as ‘innovations’: a mixed methods study in UK family practice

**DOI:** 10.1186/s12911-016-0343-y

**Published:** 2016-08-09

**Authors:** Benjamin Brown, Sudeh Cheraghi-Sohi, Thomas Jaki, Ting-Li Su, Iain Buchan, Matthew Sperrin

**Affiliations:** 1 Health eResearch Centre, Farr Institute, University of Manchester, Manchester, M13 9PL UK; 2 NIHR Greater Manchester Primary Care Patient Safety Translational Research Centre, University of Manchester, Manchester, UK; 3 Department of Mathematics and Statistics, Lancaster University, Lancaster, UK; 4 School of Dentistry, University of Manchester, Manchester, UK

**Keywords:** Clinical prediction models, Prognostic models, Risk stratification, Diagnostic models, Clinical decision support systems, Primary care information systems, Family physicians, Healthcare information technology adoption, Attitude of health personnel, Practice patterns, Clinicians

## Abstract

**Background:**

Well-designed clinical prediction models (CPMs) often out-perform clinicians at estimating probabilities of clinical outcomes, though their adoption by family physicians is variable. How family physicians interact with CPMs is poorly understood, therefore a better understanding and framing within a context-sensitive theoretical framework may improve CPM development and implementation. The aim of this study was to investigate why family physicians do or do not use CPMs, interpreting these findings within a theoretical framework to provide recommendations for the development and implementation of future CPMs.

**Methods:**

Mixed methods study in North West England that comprised an online survey and focus groups.

**Results:**

One hundred thirty eight respondents completed the survey, which found the main perceived advantages to using CPMs were that they guided appropriate treatment (weighted rank [r] = 299; maximum *r* = 414 throughout), justified treatment decisions (*r* = 217), and incorporated a large body of evidence (*r* = 156). The most commonly reported barriers to using CPMs were lack of time (*r* = 163), irrelevance to some patients (*r* = 161), and poor integration with electronic health records (*r* = 147). Eighteen clinicians participated in two focus groups (i.e. nine in each), which revealed 13 interdependent themes affecting CPM use under three overarching domains: clinician factors, CPM factors and contextual factors. Themes were interdependent, indicating the tensions family physicians experience in providing evidence-based care for individual patients.

**Conclusions:**

The survey and focus groups showed that CPMs were valued when they supported clinical decision making and were robust. Barriers to their use related to their being time-consuming, difficult to use and not always adding value. Therefore, to be successful, CPMs should offer a relative advantage to current working, be easy to implement, be supported by training, policy and guidelines, and fit within the organisational culture.

## Background

A clinical prediction model (CPM) calculates the probability of an outcome for a patient based on their individual characteristics [1]. ‘Outcomes’ may refer to future health states, such as a cardiovascular event within 10 years (e.g. QRISK [2], Framingham [3]), the presence of current health states (e.g. deep vein thrombosis in the Wells’ score [4]), or the severity of health states (e.g. lower urinary tract symptom severity in the International Prostate Symptom Score [5]). CPMs often, but not uniformly, out-perform clinicians at predicting outcomes [6, 7]. For example, in predicting outcomes after coronary bypass surgery, clinicians significantly overestimated the probability of operative mortality and length of stay on intensive care compared to a CPM [8]. However, quantitative or qualitative evaluations of the use and impact of specific CPMs in practice are often limited or non-existent [9].

In the family physician context, CPMs have been in used in some form at least since the advent of evidence based medicine in the 1990s. Pressure on UK family physicians to use CPMs has increased since use of certain CPMs has been mandated in the quality and outcomes framework [10]. CPMs are delivered in a range of forms, from being fully integrated in the clinical information system, to being available as a calculator on a website (e.g. QRISK [2]), to being a simple score that the physician calculates by hand (e.g. Wells’ score [4]).

Family Physicians deal with a wide range of problems and are faced with an ever-growing number of relevant guidelines to implement, many of which now recommend the use of CPMs. A recent literature review found that 58 CPMs are recommended across 243 guidelines relevant to family physicians, covering clinical areas including cardiovascular disease, diabetes, osteoporosis, fractures, breast cancer, infections, and mental health [11]. However, the adoption of CPMs by family physicians is low; a recent survey of United Kingdom (UK) family physicians found that as few as 2 % use specific recommended CPMs [11], with similar numbers in Germany [12]; whereas in Switzerland the number is 26 % [13], in France 32 % [14], and 35 % in Spain [15].

Some research has sought to investigate the views of family physicians regarding CPMs, which may help understand reasons for their low use; the majority of this has focused on CPMs to predict cardiovascular outcomes. Surveys of family physicians in Spain and Switzerland have revealed doubts concerning over-simplification of risk assessment, the potential for over-treatment, lack of time and computer support [13, 15]. Interviews with UK family physicians suggested there was considerable confusion regarding which CPMs were the best to use (e.g. the older versus newer CPMs), and how to use them (e.g. whether it is legitimate to use CPM scores to demonstrate the change in risk based on treatment) [16]. In Australia, family physicians suggested they used CPMs when they considered it appropriate for the patient, when they had enough time and enough experience using CPMs [17]; whereas barriers to their use included poor software, and the feeling that patients may misunderstand the communicated risk [18].

Relatively few studies have explored the views of family physicians regarding CPMs unrelated to cardiovascular disease. Two studies, in France [14] and Germany [12], explored family physicians’ views of CPMs relating to diabetes, osteoporosis, and depression (in addition to cardiovascular disease). Important themes included a lack of relevant recommendations from CPMs, a perceived lack of accuracy, interruptions to communications with patients, and a lack of time to use them [12, 14].

Although these studies provide some insight into reasons why family physicians may not use CPMs, attempts have not yet been made to theorise family physicians’ views of CPMs. We argue that CPMs should be considered complex interventions [19, 20] because they may be used in different ways by different physicians, such as to guide treatment or aid communication with patients [18]. In turn this may result in different outcomes from their use, such as a change in treatment or modification of clinician-patient communication [12, 14, 18]. The use of theory is recommended in the evaluation of complex interventions [21]. Therefore, using a theoretical framework to interpret family physician’s views of CPMs would be useful to begin to predict and generalise why certain CPMs are not used, and provide recommendations that may improve their uptake.

The aim of this study was to investigate reasons for use and non-use of CPMs by UK family physicians. It attempts to address a gap in the literature by extending the scope of enquiry beyond cardiovascular CPMs, and interpreting findings within a wider theoretical framework to provide recommendations for the future development and implementation of CPMs.

## Methods

### Study design and setting

We conducted our study in two phases, first undertaking a survey, then exploring the issues raised further with respondents in focus groups. The research was set in the North West region of England. We chose not to focus on one type of CPM during the study in order to address the need identified in the literature above to further investigate non-cardiovascular CPMs.

### Survey study

We sent an online survey to all family physician trainers and trainees in the NHS North Western Deanery in February 2014 (*n* = 956) using SoGoSurvey [22]. Questions addressed the advantages, barriers and enablers to using CPMs, which could be answered using fixed responses, ranking of (top three) preferences, and free-text (Appendix). The survey was informed by the existing relevant CPM literature identified above, in addition to our personal experience in developing, implementing and using CPMs. An initial version was piloted with six family physicians, and refined with their feedback. To encourage participation, a tablet computer was offered in a prize-draw. A reminder email to complete the survey was sent after 18 days, and the survey closed in March 2014. Results were analysed using R version 3.0.2 [23]. Where participants were asked to rank their top three preferences, each item received a *weighted rank*:

$$ r = 3\ \mathrm{x}\ \left\{\mathrm{endorsed}\ \mathrm{rank}\ 1\right\} + 2\ \mathrm{x}\ \left\{\mathrm{endorsed}\ \mathrm{rank}\ 2\right\} + 1\ \mathrm{x}\ \left\{\mathrm{endorsed}\ \mathrm{rank}\ 3\right\}. $$

The maximum score for each item per participant is therefore 3, so the overall maximum score is 3 x number of responses.

### Focus groups

Two focus groups were conducted in June 2014 by authors BB, an academic family physician with training and experience in qualitative methods, and MS, a statistician with experience in the development and implementation of CPMs. A total of 18 participants were included (nine in each group) from 14 different primary care practices from across North West England (11 female; average 8 years postgraduate medical experience, ranging from 3 to y years), and each focus group lasted one hour. Participants were recruited both from survey respondents, and professional contacts. Prior to starting the focus groups, a presentation and information sheet on the study aims were given. Written consent was obtained and participants received financial reimbursement for their time. Focus groups were semi-structured according to the following topics:

- What CPMs do participants use?
- How do they use them?
- What are the benefits of using CPMs?
- What are the disadvantages of using CPMs?
- What factors facilitate using CPMs?
- What factors are barriers to using CPMs?
- What do participants think of the survey results and free-text comments?

Facilitators provided opportunities for all participants to contribute, and discussion continued until all participants agreed there were no further issues to explore (saturation was reached). Focus groups were audio-recorded and transcribed verbatim. Thematic analysis [24] of transcripts was undertaken by authors BB and SCS (SCS is an experienced non-clinical qualitative primary care researcher) using NVivo 10 Software (QSR International). Initial inductive open coding was performed independently, and a preliminary set of themes agreed through discussion and consensus. Transcripts were then independently re-coded using this framework, and a final version of themes, definitions, and illustrative quotes agreed. Tabulation and diagrams were used to explore the relationships between themes.

## Results

### Survey study

**Fig. 1:**
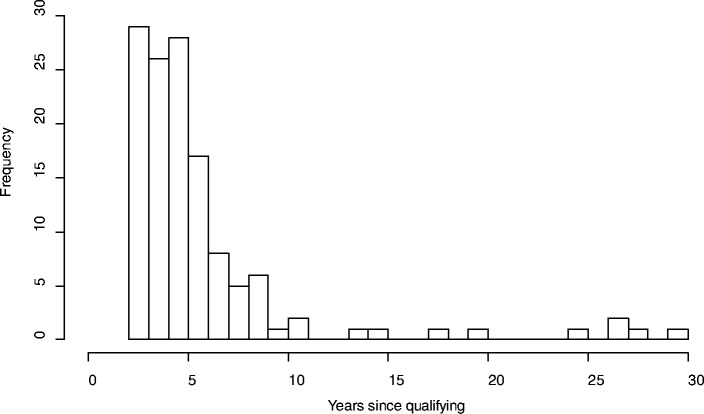
Distribution of number of years since qualifying from medical school for survey. Participants (each bar represents a single year)

The survey was completed by 138 respondents (14.4 % response rate), 64 % of whom were within 5 years of qualifying from medical school (Fig. 1). Most respondents believed family physicians either made the right amount of use of CPMs (77/138, 56 %) or should use them more (56/138, 40.6 %), with 5/138 (3.6 %) not answering. Participant responses when asked about ranking the advantages, barriers and enablers to CPM use are in Table 1. Most (125/138, 91 %) felt further training in understanding and communicating CPM outputs would be helpful. Two-thirds (90/138, 65 %) believed family physicians could regularly out-perform a CPM in predicting patient outcomes. Selected responses to open-ended question are in Table 2, linked to themes that arose from the later focus groups.

**Table 1:** Advantages, barriers and potential enablers of CPMs as endorsed by survey respondents; in weighted rank (r) order. Additionally, ‘% 1’ denotes the percentage of time an element was endorsed as the top-ranking

Advantages	r	% 1
Guides appropriate treatment	299	53
Justifies treatment decisions made	217	20
Incorporates large body of evidence	156	19
Motivates patient to make lifestyle changes	70	4
Promotes equity of treatment	62	4
Scores QOF points	21	1
Other	4	1
Barriers		
Do not have time to use during a consultation	163	24
Not relevant for use in all patients	161	19
Not integrated with electronic patient records	147	21
Do not know which risk score to use	103	12
Do not include all risk factors	92	9
Lack of link to clinical actions	63	6
Do not add to my clinical judgement	42	3
Encourage over-treatment	34	4
Encourage under-treatment	11	1
Other	8	1
Undermine my professionalism	4	1
Enablers		
Ensure good treatment decisions	133	16
Integration with electronic patient records	131	22
Quick to use	126	17
Strong link to clinical action	109	12
Add value to clinical judgement	93	12
Provides justification for clinical decisions	82	9
Easy to use	80	9
Transparency of the methods behind the risk score	32	2
Motivate patient to make lifestyle changes	27	1
Well incentivised	9	1
Other	0	0

Key – *QOF* Quality and Outcomes Framework

**Table 2:** Selected responses to open-ended survey question and links to related themes from focus groups

Response to open-ended survey question	Related themes from focus groups
Risk scores can be very useful in their place to guide treatment or investigation. The overall clinical picture can only be gained from a clinician, so they cannot replace all thought.	Perceived threat to professionalism Perceived effects on personalised care
Population risk doesn’t equal individual patient risk; these scoring systems should be used to aid discussion and communication, not as an end or decision-maker. Other scoring tools (e.g. Oxford ortho scores, IPSS, GAD-6 etc) don’t necessarily reflect ‘risk’ but are similar in their use in communication & negotiation with patients. Linking scoring tools to read codes can be useful (in the same way entering a Read code will bring up web mentor topics on EMIS for example) in assisting the clinician to utilise these tools - otherwise it’s a case of remembering the right tool and searching for it on the web.	Perceived effects on personalised care Perceived effects on communication Ease of use
Risk scores are often suggested from small pieces of research. They don’t always help guide decisions, and there is a struggle between usability and being comprehensive that many scores don’t achieve. I hate stretched acronyms (like CHADS2-VASC) where you cannot remember the components. I often use MD Calc if I need a risk score	Actionability Ease of use Knowledge of CPMs
Ultimately it is a computer generated score. It can’t replace clinical judgement however once you use it and document it, from a medico legal aspect, you have to be very confident and brave to ignore it and often this is the barrier to using it as opposed to clinical judgement in the first place. I probably use it more to add weight to my decisions.	Fear of litigation
Have seen both sides - man with a healthy lifestyle in 70s score 50 % on QRISK making him feel there was little point to his lifestyle improvements and a very unhealthy man (obese, drinker) etc who scored lowly so then thought he had justification to continue with his unhealthy lifestyle - risk scores useful when used with clinical judgement	Perceived effects on personalised care
Only useful if the basic statistical and trial data is understood by the doctor doesnt always apply to the patient/ population in front of you	Perceived effects on personalised care
Risk scores are very important, especially in general practice, but clinical judgement always reigns supreme. I like showing patients their QRISK2 score and what would happen to their risk were they to stop smoking for example. But barely-existent integration of such scores undermines their use in day-to-day consultations. Most family physician clinical systems are very poorly designed, and this is something I am planning to take up as a challenge once I complete my training and get settled.	Perceived effects on communication Ease of use
I think younger Family physicians / trainees are more aware of risk scores eg CURBS, Wells (how to use Well’s properly which is drilled into us as foundation years but Family physicians often may not know how to use properly)	Knowledge of CPMs Clinical confidence and experience

### Focus groups

**Fig. 2:**
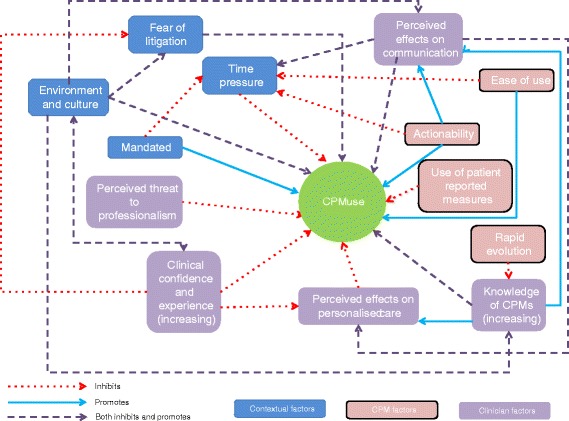
Themes and their interactions arising from focus groups

Following transcript analysis, 13 interdependent themes emerged as influential in determining family physicians’ use of CPMs. These were grouped into three domains (Fig. 2): *clinician factors* (themes: clinical confidence and experience; knowledge of CPMs; perceived effects on personalised care; perceived threat to professionalism; perceived effects on communication), *CPM factors* (themes: actionability; ease of use; use of patient-reported measures; rapid evolution) and *contextual factors* (themes: mandated; time pressure; fear of litigation; environment and culture). These are explained in more detail below.

### Clinician factors

If a participant felt confident in their clinical skills, they reported using CPMs less in that clinical area. Conversely, if participants felt less confident, they often used CPMs to compensate for a perceived lack of knowledge. Other participants used CPMs to guide clinical assessments and treatment decisions, in an effort to improve and standardise care.

**Table:** 

It depends how confident you are, in your decision making… like the PHQ-9 I am confident enough taking a mental health history and a depression history… I don’t feel that that score replaces my own clinical judgement but there would be some scores where you know I would feel that if the score told me something that I wasn’t sure of I would rely on the score more than my own because I don’t feel my own clinical acumen is good enough in that area to replace the score. *FG2R9 (family physician, medically qualified 10 years)*	

Many participants felt that more experienced doctors were less likely to use CPMs, in part due to their greater clinical confidence, and the culture in which they trained where CPMs were not as widely available.

**Table:** 

What the problem is, what has happened is, we learnt it by I don’t know 100 h a week and a few people, you know disasters happening. Now there is much more, there is much more medical senior supervision at a junior level, so there is probably less likely that the disasters will happen nowadays and you are relying on the scores… *FG2R2 (family physician, medically qualified 25 years)*	

Most participants were unaware of the evidence behind CPMs and felt that addressing this would promote their use. However, those that were aware of the evidence were cognizant of CPM limitations, which they used to inform discussions with their patients to provide personalised care.

**Table:** 

If there is somebody who is a lot older and you know the evidence for statins stops there is a cut off isn’t there and beyond that age, is it about 80, 85 you know sure it is probably going to have benefit but how much benefit is it going to have overall, what am I doing to this patient and the side effects… so it is about that conversation and saying what do you think?… and there is a difference between that person and the 40 year old as you said who overall their cumulative risk is much, much higher to them it is probably much more important. *FG2R8 (family physician trainee, medically qualified 5 years)*	

All participants believed CPMs could both facilitate and impede communication during clinical encounters. Some reported using CPMs as a segue into questions about potentially sensitive subjects like alcohol intake in the CAGE questionnaire [25] or suicidal ideation in the Patient Health Questionnaire (PHQ-9) [26]. They felt if they asked these questions in natural discourse it may adversely affect rapport, so used the CPM as an ‘excuse’ to broach these topics. Some reported avoiding difficult questions altogether by requiring patients to fill out a form comprising CPM questions. CPMs were also reported as helpful in communicating risk to patients, to inform discussion regarding their management and support shared decision-making. In this context, CPMs were found useful in both introducing statistical risk as a concept and also as a visual aid where one was provided, such as the QRISK website [27]. Conversely, some participants found CPMs could inhibit communication with patients. This was highlighted as problematic in sensitive or emotionally charged encounters where using a CPM could either interfere with the questions the doctor wanted to ask, or interrupt the consultation as the doctor’s attention was diverted to calculating or recording the CPM results.

**Table:** 

The alcohol one I found useful, purely because it feels less like nagging, so you can actually ask questions you can sort of, it’s like sexual health questions if you have a pro-forma you can… we ask these questions to everyone, and you can ask things that you wouldn’t be comfortable going through normally and you can get a lot more information out… I think the problem is and I sort of watched it on videos as well, is that they are very vulnerable and for them to start opening up and I have seen it on video and then you start tap, tap, tapping at a screen, it is partly it is that thing of all the stuff I am telling you is going onto that screen, there is that element of it, and it breaks the consultation. I have watched it and you can see patients just start to shrink back again because it’s I am just trying to tell you something really difficult, for me to talk and you are tapping away on your bloody computer it just, I hate, that is why I hate using them. *FG2R5 (family physician, medically qualified 18 years)* But it might introduce the idea in the first instance, which if you had continued intervention might sustain so like I know that in one of the family physician surgeries I worked at, you could make the QRisk into like a grid of 100 like sad faces, for like a bad outcome or a happy face to a good outcome, and I think that does help the patient because you could change like if you changed the smoking and you could suddenly have like a million more happy faces, like it seems really basic but it gives them like a pictorial representative which I think can help with the initial stages but it won’t like you said it is not going to be a sustained thing if you just expect that to carry on. *FG1R7 (family physician trainee, medically qualified 3 years)*	

Most participants reported using CPMs when communicating the clinical status of patients to secondary care when arranging a referral. Where the CPM output agreed with their clinical opinion that referral was indicated, the information was usually communicated and the CPM viewed positively. However, if the CPM’s output conflicted with their opinion, this information was often not shared voluntarily unless the discrepancy could be explained. If the receiving clinician subsequently requested the CPM’s output, participants viewed the CPM as a hindrance as it could be used to block the referral.

**Table:** 

I think because it just gives you a standard doesn’t it, that you are sort of both [referring and receiving clinician] singing from that same hymn sheet so everyone knows what they are looking at whereas before it was very descriptive for DVT [deep vein thrombosis]… and it’s, now it is well if they hit this rating then they almost have to accept them so it just makes it easier to and things like the sort of TIA [transient ischaemic attack ABCD2 score]… all those things, it just means that you, you are speaking the same language with someone on the phone who hasn’t actually got the patient in front of you. *FG2R5 (family physician, medically qualified 18 years)* I would say probably more [the] case in secondary care, partly because the pressure for the juniors there is not to admit people, and so people tend, and unfortunately be very defensive and so they will use tools that are meant to be there as tools, they will use them as tools to reject admissions. *FG2R8 (family physician trainee, medically qualified 5 years)*	

Some participants voiced concern about the risk of uncritically using or over-relying on CPMs. They felt that using CPMs was a threat to their professionalism as it over-simplified their complex clinical skills, and could be viewed as a shortcut. Furthermore, they felt that because CPMs are derived from population averages, their outputs might not be relevant to individual patients. This reduced their ability to provide personalised care, which could lead to problems such as over-medicalisation. However, through discussion, some participants managed these CPM limitations with patients as highlighted above. Consequently, CPMs were often used judiciously, which appeared to conflict with their original use to standardise care for patients.

**Table:** 

I think the problem is you see, because you are dealing with an individual, when you are using the score you are using it on an individual basis, but you are right it’s been based on a population aggregate, so therefore, you can’t just rely on the score you have to have clinical acumen as well. *FG2R2 (family physician, medically qualified 25 years)* Yes probably, we get it all the time with like Wells’ score for DVT, and people with chronic immune insufficiency so, little old ladies with huge legs, really common thing you see in family physician, and they are always immobile, erm… you know they have always got a couple of other things that would give them every day of their life they have got a Wells’ score of 3, but every time you go and see them you don’t write the Wells’ score down because you don’t, they have not got a DVT and both legs are the same but you see just, you just deliberately think well, I am not going to write that down I am just going to focus on what the clinical problem is. Because you almost, yes you don’t want to go there so you just don’t use the score. *FG1R8 (family physician trainee, medically qualified 3 years)*	

### CPM factors

Regular updates to CPMs means that participants were unlikely to have a comprehensive knowledge of all current models relevant to their practice. Almost all participants were more likely to use a CPM if its outputs were actionable – i.e. associated with a specific clinical action, such as statin prescription with QRISK [2], or referral urgency for a transient ischaemic attack with the ABCD2 score [28]. Accordingly, they were less likely to use a CPM if was perceived as less actionable e.g. those used to estimate symptom severity such as the PHQ-9 for depression [26]. As a workaround, some participants reported such CPMs to monitor change in a patient’s condition, which they could then use to guide clinical action or facilitate communication with patients or colleagues.

**Table:** 

…with more something like PHQ-9 or something, you cannot see objectively if there is much response to treatment so if they come in and they are originally 26 out of 27 and then a year later they only report themselves as a 13 you know objectively there has been progress in kind of the outcome. *FG2R7 (family physician trainee, medically qualified 3 years)*	

There was scepticism of CPMs that relied solely on patient-reported symptoms rather than physiological variables such as blood pressure. Most participants felt that patient-reported symptoms were less objective and therefore less accurate, with the potential for patients to exaggerate. This seemed to contradict how CPMs were used in situations to facilitate communication with patients and monitor their symptom severity.

**Table:** 

Well it’s the ones that you, its subjective you can’t measure them so you haven’t got a, you know a number to support, to support it as such so when you ask the patient and there is a range of responses, the Epworth [Sleepiness Scale] is a good example, you know recall is not always 100 % accurate, so they may well doze off in certain scenarios, and people may have a different interpretation what they class as low, high or moderate chance of dozing off and that would skew the score. Some people come with a score of 24 and are slim and they clearly haven’t got sleep apnoea whereas some people might have an 8 or a 9…. It is a bit of crystal ball gazing isn’t it, because the ones where you have basically got numbers like systolic blood pressure, LDL cholesterol whatever the ones where you have got actual numbers, that, that it trumps your acumen frankly because you know you can’t, that is done on an epidemiological average study of how likely this chap is to have a vascular event in the next 10 years or whatever, you can’t crystal ball gaze the ones where it is about the, the here and now and there is lots of subjectivity within the score *FG2R6 (family physician, medically qualified 10 years)*	

Almost all participants reported they were more likely to use CPMs that were easy and quick to use, for example those where the electronic health record (EHR) automatically performed the calculation. This was enhanced if the EHR reminded them to complete the CPM during the clinical encounter.

**Table:** 

I think it is a major point that if something does flash up on [an EHR] and you have to fill it in, then you will as opposed to if you have to search through something and it says click on this link and it takes you to like four webpages then it won’t get filled in. I think in just the reality of the situation. *FG1R2 (family physician trainee, medically qualified 5 years)*	

### Contextual factors

One of the strongest factors nearly all participants reported determining their CPM use was whether or not they were mandatory. This could be through national clinical guidelines, local policies or the Quality and Outcomes Framework (QOF; the national pay-for-performance scheme in UK primary care) [29]. The issue of mandate was augmented by professional norms, specifically whether there was a culture of using CPMs by colleagues. A contrast between primary and secondary care was noted, where participants felt CPMs were used more frequently in the latter. This was felt by participants to partly explain why less experienced family physicians reported using CPMs more readily (as described under ‘clinician factors’) given they had more recently worked in secondary care compared with their more senior colleagues.

**Table:** 

I think I mean I use them but probably I only use them because we are told to use them in guidance, CHADS2 VASC you are told to use it in AF [atrial fibrillation] guidance you know… QRISK we have to use it, to justify decision making and statins and things like that, Epworth [Sleepiness Scale] I was told we have to use Epworth if we are referring to the sleep clinic. *FG2R9 (family physician, medically qualified 10 years)* I think that is definitely truer at the more junior level, in these more process driven environments like A&E because I am [a trainee family physician] and I remember in A&E there are sort of proformas almost based on risk scores for assessing certain conditions and that does help you almost learn the questions that you should be asking to assess someone and if it does change as you get more senior and move into general practice it is a lot less process driven and it is a lot more practitioner driven, then it very much will depend on what your own personal awareness is of the scores, whether you actually choose to use one rather than kind of being forced to use one, because that is like the rules of the department that you might be working in *FG1R2 (family physician trainee, medically qualified 5 years)*	

Fear of litigation determined whether some participants used CPMs. When the CPM outputs indicated low risk for a potentially important outcome (e.g. pulmonary embolism) this was sometimes documented in patient’s medical records to demonstrate that a relevant clinical option had been considered and validly excluded. Conversely, if a CPM output was anticipated to indicate high risk and hence further action, the CPM was not used if this anticipated output was discordant with the participant’s clinical judgment. This was similar to participants not using CPMs when they reduced their perceived ability to provide personalised patient care. Fear of litigation was more pronounced in more junior participants perhaps due to lower confidence, and influenced by the culture of the participants’ working environment.

**Table:** 

In some ways if you think they don’t really have clot or a DVT you are kind of using it really for medico-legal point of view because you don’t actually believe this patient does but just in case you know they do ever turn out to have a clot or PE then hopefully this will now show that I kind of considered all the other factors. *FG2R3 (family physician trainee, medically qualified 5 years)*	

Most participants reported lack of time as a key reason for not using CPMs. This was mitigated if the CPM was quicker and easier to use, if the CPM’s output was actionable or could facilitate communication with the patient or a colleague, or if use of the CPM was mandatory. Primary care was felt to have more time pressure than secondary care.

**Table:** 

I mean it’s ultimately what you are going to get out of it, in a 10 min consultation so if you are going to use it for those things… because you have to do it for QOF because you have to do it to augment a referral or get the referral in, if you have to do it for persuasion… you know you will use that in a 10 min consultation but if it doesn’t confer you any benefit to do that… with time constraints you are going to go to your, if you like less measured, softer clinical skills in how you find the patient. *FG2R6 (family physician, medically qualified 10 years)*	

## Discussion

### Summary and interpretation of findings

This mixed methods study explored factors influencing CPM use as reported by UK family physicians. Our survey identified the main advantages to using CPMs were: to guide appropriate treatment, to justify treatment decisions, and to incorporate a large evidence base. The most endorsed barriers were: lack of time, irrelevance to some patients, and poor integration with EHRs. Focus groups found 13 themes to explain CPM use covering three domains: clinician factors, CPM factors and contextual factors. Many of these themes were interdependent (Fig. 2), highlighting the tensions that clinicians face, such as providing evidence based care that is also personalised and informed by clinical judgement, or to balance the use of CPMs between facilitating and inhibiting communication with patients.

Diffusion of Innovations theory in health service organisations states that whether innovations are adopted is determined by features of the innovation, the adopters, and wider context [30]. These categories are analogous to the broad thematic domains of CPM, clinician, and contextual factors derived from our focus groups, hence we used Diffusions of Innovations theory to frame our results.

Many of the reasons for use and non-use of CPMs we found can be explained by the concept of ‘relative advantage’ [30]. Diffusion of Innovations theory posits that innovations must have a clear unambiguous advantage over usual ways of working to be readily adopted. This was demonstrated in the survey, where the most endorsed advantages of CPMs were to guide and justify appropriate treatment. This was also seen in focus groups where participants reported using CPMs in situations where their clinical knowledge was weak, and where CPMs were felt to enhance communication with colleagues and patients. However, our results also demonstrated that use of CPMs may be more complex than this. The potential advantage may be more ambiguous, such as where they served a role in medico-legal protection for the physician. A perceived lack of relative advantage was demonstrated in the survey with the finding that most (90/138, 65 %) respondents believed that family physicians could regularly out-perform CPMs in predicting patient outcomes. On the other hand, a lack of link to a clinical action, and irrelevance to a patient were some of the highest ranked barriers to using CPMs. Similarly, in the focus groups, participants reported using CPMs less, or not at all, when it was felt that CPMs did not provide an advantage, or imposed a relative disadvantage, such as interfering with doctor-patient communication, or the provision of personalised care.

Further explanations of our results by Diffusion of Innovations theory relate to the constructs of ‘complexity’, ‘compatibility’, ‘dedicated time and resources’, ‘political directives’ and ‘participants’ concerns’ [30]. With respect to complexity, innovations that are perceived by clinicians as simple to use are more readily adopted. This was particularly true if they were automated, and physicians were reminded to use them within the EHR; this feature was also highly endorsed by survey respondents.

Innovations that are compatible with the intended adopters’ values, norms, and perceived needs are more readily adopted. This is congruous with our finding of the importance of culture of use of CPMs within a family physician practice in determining whether or not they are used.

A lack of time is recognised as an important determinant of a whether an innovation is adopted (‘dedicated time and resources’). This was the highest ranked barrier to using CPMs in the survey, which was further emphasised within the focus groups with regards to the lack of time for using CPMs within a clinical consultation.

Political directives are acknowledged in Diffusion of Innovations theory as effective drivers for organisations to adopt innovations. This is consistent with our findings that clinical guidelines and local policies mandating the use of CPMs are effective at increasing their uptake.

The Concerns Based Adoption model in Diffusion of Innovations theory suggests that in the pre-adoption phase, intended users must be aware of the innovation and have sufficient information about what it does and how to use it [30]. This explains why nearly all (125/138, 91 %) of survey respondents felt they needed further training in the use of CPMs. It also explains why focus groups revealed that regular updates to CPMs are a barrier to their use, because it reduces family physicians’ familiarity with their correct application.

### Comparison with existing literature

Other literature supports the consideration of CPMs as innovations, which should be viewed in a wider theoretical framework [19]. The notion of *disruptive* innovation, creating a new ‘market’ in clinical decision making may also apply [31]. The ‘disruption’ emphasises the activation energy required to change practitioners from a largely CPM-absent to a CPM-supported decision making norm [32].

Using this theoretical lens, our findings can also be compared with the existing literature into family physician’s use of CPMs. Barriers to using CPMs found in studies regarding concerns of over-simplification of risk assessment and a perceived lack of accuracy [12–15] relate to the notion of relative advantage, which suggests that if CPMs are not perceived as more effective than the current way of working they will not be adopted. The feeling that CPMs may lead to over-treatment of patients [13, 15] can be viewed as a manifestation of ‘compatibility’ in that family physicians strive to provide personalised care. If CPMs are perceived to interfere with this, they will not be used. Finally, findings that family physicians expressed confusion regarding which CPMs to use and how to use them [16], relate to the Concerns Based Adoption model and the prerequisite that intended adopters should have sufficient information about what an innovation does and how they should use it for effective uptake.

Some of our findings are supported by other studies of family physician views of CPMs. For example, our finding that a lack of time to use CPMs and software support are important barriers to their use have been demonstrated in Spain, Switzerland, France, Germany, and Australia [12–15, 17]. Our notion of actionability as a facilitator to CPM use is supported by observations in Germany, where a lack of relevant lifestyle-related recommendations for healthy patients prevented family physicians from using CPMs [12]. Our finding that using CPMs may impede (as well as improve) communication with patients and colleagues is mirrored by a study in France where family physicians stated they did not use CPMs if they interrupted the flow of the clinical consultation, though did use them to convey information in specialist referrals [14]. An interesting insight into actionability is offered by a recent study of a specific CPM in the UK [33], which highlighted another way in which a CPM may not be actionable – where a recommended service is not available.

Our study also makes new contributions to the literature. We found that UK primary care clinicians use CPMs for purposes not previously reported, such as communicating with other clinicians, for medico-legal purposes, and to legitimise asking patients difficult questions. We also identified new CPM features that may influence their use, such as the potential barrier of using patient-reported data to generate CPM outputs, and the potential enabler of linking CPM results to a defined clinical action (actionability). Our findings also suggest a complexity and tension between different factors that influence CPM use that has not previously been reported (Fig. 2). This arguably contradicts the overly simplistic application of terms such as ‘barriers’ and ‘enablers’ to the use of CPMs, as has been previously observed in other studies of evidence based medicine in UK primary care [34].

### Strengths and limitations

Using focus groups in addition to a survey allowed deeper exploration of issues identified within the survey, and revealed complexities in family physician’s views of CPMs. We did not focus on participant views of CPMs in a particular clinical area, such as cardiovascular disease, to enable a wider range of views and characteristics of CPMs to be explored. However, our general focus may have prevented a more in-depth analysis of issues with specific types of CPM. Although useful for understanding participant views, both surveys and interviews are susceptible to recall bias. For example, there may be additional reasons that physicians use (or do not use) CPMs that are only apparent following observation by someone else.

Our survey response rate was low (14.4 %), though in similar studies the response rate could not be calculated [11], and where it has been reported is comparable to our own [35]. Nevertheless, a low response rate may have biased our survey towards participants that are interested in CPMs and were therefore more motivated to complete the survey. This may have resulted in more extreme views than the wider family physician population. Moreover, the survey was sent to a list of trainers and trainees, hence clinicians who were neither of these were not reached, and there was also a bias towards newly qualified clinicians. As we used the survey to also recruit participants for focus groups this may also have influenced their findings, though in contrast to surveys, having respondents with an interest in the topic matter can be advantageous in capturing diversity of opinions [36]. Finally, we limited our study to one region of England, so the views reported here may not be representative of the wider family physician population.

### Implications for practice and research

Our findings, and their interpretation within a wider theoretical framework, provide new insights into how primary care clinicians use CPMs, which are relevant for the future development and implementation of CPMs. Table 3 summarises best practices for development and implementation of CPMs in primary care. Primarily, and as also argued by others [19], we suggest that CPMs should be considered as innovations, driven by ongoing science and technology, which are applied in complex clinical environments. To ensure CPMs are successfully adopted, attention should be paid to the factors relating to the CPM itself, the clinicians intended to use it, and the context. Any new CPM that is intended for implementation should thus be thoroughly evaluated using both quantitative and qualitative methods to ensure that their inherent complexity is captured. Such evaluations are, at present, usually inadequate [9].

**Table 3:** Best practices for CPM development and implementation in primary care

*CPM factors*
• The CPM should have a relative advantage to current ways of working. This could be achieved in the following ways: ○ Focusing on areas where there is a perceived lack of clinical knowledge or risk of medical litigation ○ Linking outputs to clinical actions ○ Provide ways to enhance, not impede, communication with patients and colleagues ○ Provide the ability to monitor symptom severity • CPMs should be as simple and easy to use as possible. This could be achieved by using only routinely measured risk factors and markers to calculate the score, then auto populating the result within existing clinical information systems.
*Clinician factors*
• Clinicians must be made aware of new CPMs, and if existing ones are updated • Information or training on how to use the CPM should be provided (consistent with providing personalised care), which should also highlight the advantages of its use.
*Contextual factors*
• The CPM should be compatible with the pervading culture of the organisation in which it is being implemented. • CPM use should be recommended in clinical guidelines or local policies. • Dedicated time and resources should be provided to use CPMs. • Education and training in the use of CPMs should be available.

#### CPM factors

Particular consideration should be given to the ‘relative advantage’ of the CPM, as it is more likely to be used if it seemed to be a more effective way of working than the status quo. Examples of how this could be achieved as demonstrated by our results are to focus CPM development in clinical areas where there is a perceived lack of clinical knowledge, or to ensure that their outputs are linked to clinical actions, as has also been highlighted in other studies [12]. CPMs should be as simple and easy to use as possible. Our results suggest this may be achieved by integrating them with clinical information systems, including automatic extraction of relevant input data and recording of (actioned) model estimates. This automation could negate problems when CPMs are revised. Particular care would be needed to handle a situation where the revision means a patient’s risk crosses a clinically significant boundary – including alerting the clinician to this fact and justifying why the revised evidence now supports a new proposed action.

#### Clinician factors

As demonstrated in our findings, and in those of related studies [16], clinicians must be made aware of new CPMs, and if existing ones are updated. They should also be provided with enough information regarding what it does and how to use it [30]. This could be achieved through targeted training, which could also address the misconception highlighted in our results that most family physicians (90/138, 65 %) believe they can out-perform a CPM in predicting patient outcomes [6, 7]. Guidance on how CPMs could, should (and should not) be used, along with their potential benefits, in ways consistent with the provision of personalised care should be available. This should include acknowledging their limits in specific patient groups, or highlighting the risks of over-treatment if used uncritically. Such guidance could recommend using model outputs as a basis for communicating risk and exploring treatment options with patients (both medication and lifestyle-related), as some of our participants demonstrated. This would go some way to address the concerns demonstrated by our participants regarding tensions between the use of CPMs and the provision of personalised care, in addition to those highlighted in other studies about over-simplification of risk and over-treatment [12–15]. Providing this information would also address the issue of perceived ‘relative advantage’.

#### Contextual factors

CPMs are unlikely to be used in organisations where they are incompatible with the pervading culture, such as attitude towards evidence-based medicine, or there is a disinterest in the clinical condition the CPM addresses. This may be counteracted by relevant education or training. ‘Political directives’ such as making CPMs mandatory in clinical guidelines or local policies may increase their uptake. Lack of time is such an important issue in primary care as demonstrated in both our findings and those of others [12–15, 17] that both dedicated resources should be provided to use them, and they should be as easy and quick to use as possible (as described above).

The influence of some factors found in our study is less clear and warrants further investigation in future research. Using patient-reported measures in CPMs may reduce acceptance by clinicians, but conversely may help facilitate communication with patients. Mandating CPMs may increase their use, but may adversely affect the clinician’s sense of professionalism. Clinician seniority and organisational attitude toward CPMs may also be important. In particular, there was a suggestion that a clinician who perceives themselves as more experienced in a clinical area may rely less on a CPM than a less experienced clinician. These hypotheses could be tested in future studies of family physicians from other contexts and using different study designs. For example, an interesting avenue for further research may involve the ethnographic observation of how CPMs are used (or not used) in practice, and how they impact on patient care, rather than relying on self-reports of physicians.

## Conclusions

We investigated the reasons for use and non-use of CPMs by UK family physicians through a survey and focus groups. Our findings suggest important factors relate to features of the CPM itself, the clinician, and the context in which it is used. Based on this, we suggest best practices for the development and implementation of CPMs, summarised as follows.

The CPM should offer a relative advantage to current ways of working, and be easy and quick to use, and well integrated in the clinical workflow. Clinicians should be trained in the use of CPMs, and their use should be promoted. CPMs should be recommended in guidelines and policy, and be integrated in the clinical culture.

Our study also found that CPMs were used by participants in ways that had previously not been reported, such as communication with other doctors, medico-legal purposes, and legitimisation of difficult discussions with patients. It also identified new CPM characteristics that influence their use, such as the use of patient-reported data and actionability of results. We considered our findings within the framework of Diffusion of Innovations theory for health service organisations, which provided further insights and a structure with which to translate our results and those from other relevant studies into practical recommendations for future CPM development and implementation in primary care.

## Abbreviations

CPM, clinical prediction model; EHR, electronic health record; QOF, quality and outcomes framework; UK, United Kingdom

## Appendix

### Final survey questions

As a practising family physician or trainee in the North West you are invited to take part in a short survey regarding what you think about clinical risk scores such as QRISK, CHA2DS2-VASc, PHQ-9, Epworth Sleepiness Scale, or the Wells’ Score. The results from this survey will be used to make better risk scores. We use risk scores every day to help us manage patients, so undertaking this survey will help improve care for patients and make your life easier too.

1. Do you think family physicians make enough use of risk scores?
  - Should be used less
  - About right
  - Should be used more
2. What are the main **advantages** of using risk scores (rank all answers that apply)
  - Scores QOF points
  - Guides appropriate treatment
  - Motivates patient to make lifestyle changes
  - Justifies treatment decisions made
  - Incorporates large body of evidence
  - Promotes equity of treatment
  - Other (specify):_____________________________
3. Which of the following **barriers** prevent you from using risk scores more (rank all answers that apply)
  - Do not have time to use during a consultation
  - Do not add to my clinical judgement
  - Not relevant for use in all patients
  - Not integrated with electronic patient records
  - Lack of link to clinical actions
  - Encourage over-treatment
  - Encourage under-treatment
  - Do not include all risk factors
  - Undermine my professionalism
  - Do not know which risk score to use
  - Other (specify):_____________________________
4. Which of the following **benefits** (would) encourage you to make more use of risk scores (rank all answers that apply)
  - Well incentivised
  - Quick to use
  - Easy to use
  - Add value to clinical judgement
  - Integration with electronic patient records
  - Strong link to clinical action
  - Ensure good treatment decisions
  - Motivate patient to make lifestyle changes
  - Provides justification for clinical decisions
  - Transparency of the methods behind the risk score
  - Other (specify): _______________________
5. Would further training in understanding and communicating the results of risk scores be helpful?
  - Yes, and I would definitely attend such training
  - Yes, but I would be unlikely to find time to attend
  - No
6. Do you believe that a family physician could out-perform a risk score in predicting future outcomes for an individual patient?
  - Yes, almost always
  - Yes fairly regularly
  - No
7. Are there any other comments you wish to make about the use of risk scores? Please do so below…
8. Are you (tick all that apply):
  - Family physician trainee
  - Salaried family physician
  - Locum family physician
  - Family physician Partner
9. Years since qualified from medical school (specify)

## References

[1] Steyerberg EW. Clinical Prediction Models: A Practical Approach to Development, Validation, and Updating. New York City, USA: Springer; 2008

[2] Hippisley-Cox J, Coupland C, Vinogradova Y, Robson J, May M, Brindle P (2007). Derivation and validation of QRISK, a new cardiovascular disease risk score for the United Kingdom: prospective open cohort study. BMJ.

[3] D’Agostino RB, Vasan RS, Pencina MJ, Wolf PA, Cobain M, Massaro JM, Kannel WB (2008). General cardiovascular risk profile for use in primary care: the Framingham heart study. Circulation.

[4] Wells PS, Hirsh J, Anderson DR, Lensing AW, Foster G, Kearon C, Weitz J, D’Ovidio R, Cogo A, Prandoni P, Girolami A, Ginsberg JS (1998). A simple clinical model for the diagnosis of deep-vein thrombosis combined with impedance plethysmography: potential for an improvement in the diagnostic process. J Intern Med.

[5] Barry MJ, Fowler FJ, O’Leary MP, Bruskewitz RC, Holtgrewe HL, Mebust WK, Cockett AT (1992). The American Urological Association symptom index for benign prostatic hyperplasia. The Measurement Committee of the American Urological Association. J Urol.

[6] Toll D, Janssen K, Vergouwe Y, Moons K (2008). Validation, updating and impact of clinical prediction rules: a review. J Clin Epidemiol.

[7] Adams ST, Leveson SH (2012). Clinical prediction rules. BMJ.

[8] Ghotkar SV, Grayson AD, Fabri BM, Dihmis WC, Pullan DM (2006). Preoperative calculation of risk for prolonged intensive care unit stay following coronary artery bypass grafting. J Cardiothorac Surg.

[9] Steyerberg EW, Moons KG, van der Windt DA, Hayden JA, Perel P, Schroter S, Riley RD, Hemingway H, Altman DG (2013). Prognosis research strategy (PROGRESS) 3: prognostic model research. PLoS Med.

[10] Roland M (2004). Linking physicians’ pay to the quality of care--a major experiment in the United kingdom. N Engl J Med.

[11] Plüddemann A, Wallace E, Bankhead C, Keogh C, Van der Windt D, Lasserson D, Galvin R, Moschetti I, Kearley K, O’Brien K, Sanders S, Mallett S, Malanda U, Thompson M, Fahey T, Stevens R (2014). Clinical prediction rules in practice: review of clinical guidelines and survey of GPs. Br J Gen Pract.

[12] Müller-Riemenschneider F, Holmberg C, Rieckmann N, Kliems H, Rufer V, Müller-Nordhorn J, Willich SN (2010). Barriers to routine risk-score use for healthy primary care patients: survey and qualitative study. Arch Intern Med.

[13] Eichler K, Zoller M, Tschudi P, Steurer J (2007). Barriers to apply cardiovascular prediction rules in primary care: a postal survey. BMC Fam Pract.

[14] Sarazin M, Chiappe SG, Kasprzyk M, Mismetti P, Lasserre A (2013). A survey of French general practitioners and a qualitative study on their use and assessment of predictive clinical scores. Int J Gen Med.

[15] Elustondo SG, Aguado PN, de La Rasilla Cooper CG, Manzanet JP, Sendín DS (2013). Cardiovascular risk tables: opinion and degree of use of Primary Care doctors from Madrid, Spain. J Eval Clin Pract.

[16] Liew SM, Blacklock C, Hislop J, Glasziou P, Mant D (2013). Cardiovascular risk scores: qualitative study of how primary care practitioners understand and use them. Br J Gen Pract.

[17] Bonner C, Jansen J, McKinn S, Irwig L, Doust J, Glasziou P, Hayen A, McCaffery K (2013). General practitioners’ use of different cardiovascular risk assessment strategies: a qualitative study. Med J Aust.

[18] Torley D, Zwar N, Comino EJ, Harris M (2005). GPs’ views of absolute cardiovascular risk and its role in primary prevention. Aust Fam Physician.

[19] Noble D, Mathur R, Dent T, Meads C, Greenhalgh T. Risk models and scores for type 2 diabetes: systematic review. BMJ. 2011;343:d7163. 10.1136/bmj.d7163 PMC3225074 22123912

[20] Craig P, Dieppe P, Macintyre S, Michie S, Nazareth I, Petticrew M. Developing and Evaluating Complex Interventions: New Guidance. Medical Research Council, UK London; 2008

[21] Moore G, Audrey S, Barker M, Bonell C, Hardeman W, Moore L, Cathain AO, Tinati T, Wight D, Baird J (2014). Process evaluation of complex interventions: UK Medical Research Council (MRC) guidance.

[22] SoGoSurvey [http://www.sogosurvey.com/]

[23] “R Core Team”. R: A Language and Environment for Statistical Computing. Vienna, Austria: R Foundation for Statistical Computing: 2013.

[24] Ritchie J, Spencer L. Qualitative data analysis for applied policy research. Qual Res Companion. 2002;573:305-329.

[25] Ewing JA (1984). Detecting alcoholism. JAMA.

[26] Kroenke K, Spitzer RL, Williams JB (2001). The PHQ-9: validity of a brief depression severity measure. J Gen Intern Med.

[27] QRISK2 - 2015 risk calculator [http://www.qrisk.org/]

[28] Rothwell PM, Giles MF, Flossmann E, Lovelock CE, Redgrave JNE, Warlow CP, Mehta Z (2005). A simple score (ABCD) to identify individuals at high early risk of stroke after transient ischaemic attack. Lancet (London, England).

[29] Quality and Outcomes Framework [http://www.hscic.gov.uk/qof]

[30] Greenhalgh T, Robert G, Macfarlane F, Bate P, Kyriakidou O (2004). Diffusion of innovations in service organizations: systematic review and recommendations. Milbank Q.

[31] Christensen C, Grossman J, Hwang J. The innovator’s prescription: a disruptive solution for health care. New York City, USA: McGraw-Hill Professional; 2009

[32] Buchan I (2011). Informatics for healthcare systems. Healthcare management.

[33] Porter A, Kingston MR, Evans BA, Hutchings H, Whitman S, Snooks H (2016). It could be a “Golden Goose”: a qualitative study of views in primary care on an emergency admission risk prediction tool prior to implementation. BMC Fam Pract.

[34] Checkland K, Harrison S, Marshall M (2007). Is the metaphor of “barriers to change” useful in understanding implementation? Evidence from general medical practice. J Health Serv Res Policy.

[35] Dallongeville J, Banegas JR, Tubach F, Guallar E, Borghi C, De Backer G, Halcox JPJ, Massó-González EL, Perk J, Sazova O, Steg PG, Artalejo FR (2012). Survey of physicians’ practices in the control of cardiovascular risk factors: the EURIKA study. Eur J Prev Cardiol.

[36] Patton M, Patton M (1990). Purposeful sampling. Qualitative evaluation and research methods.

